# Coronary artery disease-associated immune gene *RBP1* and its pan-cancer analysis

**DOI:** 10.3389/fcvm.2023.1091950

**Published:** 2023-03-09

**Authors:** Yumian Wang, Li Zhang, Han Chen, Juan Yang, Yun Cui, Hong Wang

**Affiliations:** Department of Cardiology, the Affiliated Cardiovascular Hospital of Kunming Medical University (Fuwai Yunnan Cardiovascular Hospital), Kunming, China

**Keywords:** coronary artery disease, cancer, immune infiltration, retinol-binding protein 1 (RBP1), GEO

## Abstract

**Purpose:**

To identify immune-related biomarkers in coronary artery disease (CAD), investigate their possible function in the immunological milieu of tumors, and initially investigate the mechanisms and therapeutic targets shared by CAD and cancer.

**Methods:**

Download the CAD-related dataset GSE60681 from the GEO database. GSVA and WGCNA analyses were performed based on the GSE60681 dataset to identify the modules most pertinent to CAD, identify candidate hub genes and finally intersect the genes associated with immunity downloaded from the import database to find the hub genes. The GTEx, CCLE, and TCGA database were used to examine the expression of the hub gene in normal tissues, tumor cell lines, tumor tissues, and different tumor STAGES. One-factor cox and Kaplan-Meier analyses were performed to explore the prognosis of hub genes. Hub gene methylation levels in CAD and cancer were analyzed in the diseaseMeth 3.0 and ualcan databases, respectively. R package CiberSort processed the GSE60681 dataset to assess immune infiltration in CAD. TIMER2.0 evaluated hub genes with pan-cancer immune infiltration. The hub genes were analyzed for drug sensitivity and correlation with TMB, MSI, MMR, cancer-related functional status, and immune checkpoints in different tumors. Finally, GSEA was carried out on the crucial genes.

**Results:**

WGCNA were used to pinpoint the green modules that were most closely related to CAD and intersections with immune-related genes were taken to remember the pivotal gene *RBP1*. *RBP1* is hypermethylated in CAD and multiple cancers. Its expression levels in different cancers were associated with poor prognosis of cancer, with significant expression levels at higher stages of cancer staging. The immune infiltration results showed that *RBP1* was closely associated with CAD and tumor-associated immune infiltration. The results indicated that *RBP1* was strongly correlated with TMB, MSI, MMR, cancer-associated functional status, and immune checkpoints in various cancers. *RBP1* was related to the sensitivity of six anticancer drugs. GSEA showed *RBP1* was associated with immune cell activation, immune response, and cancer development.

**Conclusion:**

*RBP1* is a pivotal gene associated with immunity in CAD and pan-cancer and may mediate the development of CAD and cancer through immunity, making it a common therapeutic target for both.

## Introduction

Coronary artery disease (CAD) is a chronic inflammatory cardiovascular disease caused by structural or functional abnormalities of the coronary arteries, resulting in myocardial ischemia and hypoxia (1). Various clinical manifestations of CAD include myocardial infarction, stable and unstable angina, and sudden cardiac death (2). Although the incidence of coronary heart disease has declined in developed countries recently, it is still one of the main reasons of death with wide variations in the incidence of CAD in developing countries (3, 4). In addition, the incidence, prevalence, and mortality of cancer are increasing, and it is the leading causes of human mortality worldwide (5, 6). The cardiovascular disease shares the same risk factors as cancer, such as smoking, obesity, and diabetes. It has been shown that cardiovascular disease increases the risk of developing cancer, and in some cancer patients, it is the main cause of death (7) and CAD is the most common complication for cancer patients (8). Cardiovascular health (CVH) has recently been identified by the American Heart Association (AHA) as playing a crucial role in the prevention of CAD and cancer (9). However, how CAD and cancer are related is still unknown. Therefore, it is of great research value to find the potential signaling pathways between CAD and cancer and to explore their common therapeutic targets.

CAD is a chronic inflammatory cardiovascular disease that is primarily due to the formation of atherosclerotic plaques within the vessel wall (10). It has been demonstrated that different immune cells and immunological-mediated inflammatory responses play a role in how atherosclerotic plaques develop (11). Among them, INF-*γ*, IL-17 and IL-21, and B-cell inflammatory genes are significantly increased in people with CAD (12), and IL-17a promotes the epithelial to mesenchymal transition in gastric cancer cells through JAK2/STAT3, exacerbating the development of gastric cancer (13). Immunosuppressive effects of B cells on tumors in the tumor microenvironment (TME) inhibit cancer development (14). In mice studies, B2 lymphocytes were found to produce interferon (IFN) and pathogenic IgG and activate T cells as atherogenic immune cytokines (15). CD8+ T cells are cytotoxic and can kill tumor cells, and T helper 1 (Th1) cells can promote the appreciation of CD8+ T cells and NK cells by secreting IL-2 and IFN-*β*. The aggregation of these immune cells in TME suggests a good prognosis (16). In CAD development, CD8+ T lymphocytes play two distinct roles. On the one hand, inflammatory cytokines produced by CD8+ T cells boost the inflammatory response and aid in vascular atherosclerosis progression. In contrast, their cytotoxic effect on antigen-presenting cells and the presence of a subpopulation of cells with regulatory effects on CD8+ T cells suppress the immune response and protect against atherosclerosis (17). CAD and cancer share the same immune link, and the development of CAD and cancer is influenced by immune cells and the immunological responses they mediate. Immunotherapy has been used to better effect in the treatment of cancer, and this may be of value in the treatment of coronary heart disease.

Bioinformatics uses mathematics, biology, and computer science to mine. It analyzes data and information from medical and life sciences to explore the underlying mechanisms of disease occurrence *via* computer software, networks, and other tools (18). GSE60681 dataset associated with CAD from the Gene Expression Omnibus (GEO) database were downloaded to explore the association between immunity in CAD and cancer. Enrichment of the samples was assessed using Gene Set Variance Analysis (GSVA), followed by Weighted Gene Co-Expression Network Analysis (WGCNA) analysis to find the modules most significantly associated with CAD and to identify candidate hub genes. Next, immune-related genes were downloaded from the import database while taking intersections with candidate hub genes from CAD-related modules to identify hub genes. Finally, we assessed its expression levels in normal and different cancer tissues and further performed the hub gene's survival analysis and methylation analysis. In addition, an immune infiltration analysis of CAD and pan-cancer were conducted to explore the correlation among the hub genes, immune cells and cancer. The results may have implications for developing novel immunotherapies for CAD and cancer. Furthermore, the results can open up fresh perspectives on immunotherapies to lower the incidence of CAD in cancer patients.

## Materials and methods

### Data acquisition and pre-processing

GSE60681 dataset associated with CAD were downloaded from the GEO database for analysis. When multiple probes identify the same gene, the examination with the highest significant expression value will be selected by the GPL4133 platform's annotation data. The data were normalized using the normalized between arrays() method from the “limma” R package.

### Gene set variation analysis

GSVA transforms the expression matrix of genes between various samples into the expression matrix of gene sets between samples to determine whether different pathways are enriched between other models. Firstly, the hallmark gene set information from the minder R package was downloaded and then GSE60681 was analyzed using the GSVA R package to obtain the GSVA score of the corresponding pathway for each sample. Finally, the pathway differences between CAD and the “limma” R package were used to evaluate control groups.

### Weighted gene co-expression network analysis

WGCNA is a tool suitable for performing complex data analysis of multiple samples. By calculating expression relationships between genes, we identify gene collections (modules) with similar expression patterns, resolve associations between gene collections and sample phenotypes, regulate mapping networks between genes in gene collections, and identify essential regulatory genes. To screen for differentially expressed genes (DEGs), each gene's standard deviation was calculated and the top 5,000 genes with the most significant standard deviations were selected. Based on the GPL4133 platform's microarray dataset, a co-expression network of DEGs utilizing the R package “WGCNA” was built. When using 0.85 as the correlation coefficient threshold, a soft threshold power of 14 and a minimum number of genes in the selection module of 30 were chosen, and 0.2 was taken as the cutting height criterion to integrate any potentially similar modules. Candidate hub genes were deemed to have gene significance (GS) >0.15 and module membership (MM) values >0.8 in the modular trait correlation study, indicating that candidate hub genes were strongly related to clinical traits. To further understand the function of DEGs in the modules most relevant to CAD, gene ontology (GO) and Kyoto Encyclopedia of Genes and Genomes (KEGG) pathway enrichment analyses were performed using the meta-scale database for genes in necessary modules to identify potential mechanisms and biological pathways.

### Identification and expression level analysis of immune-related hub genes

Given that immunity plays a vital role in CAD, the import database's list of immune-related genes were downloaded and the immune-related genes with the candidate hub genes were interested as well as the immune-related hub genes were identified. In the GSE60681 dataset, the hub gene's expression profile data for the CAD and control groups were retrieved and the hub gene's expression level were examined. Standard tissue samples, tumor cell lines, and tumor samples were obtained from the GTEx, CCLE, and TCGA databases followed by analyzing the expression levels of hub genes in normal tissues, tumor cell lines, and tumor tissues based on sample information from the GTEx database, CCLE database, and TCGA database, respectively. Also, standard samples from the GTEx database were combined with tumor samples from the TCGA database to analyze the differential expression levels of hub genes in normal and tumor tissues. Based on the TCGA database of paired normal and tumor tissues, we also evaluated the various expression levels of hub genes in paired normal and tumor tissues.

### Predictive analysis of hub genes

According to survival data from patients in the TCGA database, the prognostic impact of the hub gene was investigated by using univariate cox analysis and KM analysis, including overall survival (OS), progression-free interval (PFI), and disease-specific survival (DSS).

### Methylation analysis and stage characterization of hub genes

To examine how methylation affects hub gene expression, the hub genes' methylation levels in atherosclerosis was analyzed using the disease myth 3.0 database. The methylation levels of hub genes in tumors were also analyzed using the ualcan database. A *P*-value of less than 0.05 was considered statistically significant. The hub genes' expression levels at various tumor stages were ecaluated by using the stage information of TCGA database patients.

### Immuno-infiltration analysis of CAD

To create the immune cell infiltration matrix, the GSE60681 expression matrix data was analyzed by using the R package CiberSort. The percentage of immune cell infiltrations of 22 was displayed using cumulative histograms generated using the ggplot2 R package. The connection between immune cells was analyzed using the complot R package. Differences in immune cell infiltration between the CAD and control groups were analyzed and visualized using the ggplot2 R package. We performed spearman correlation analysis on hub genes and immune cells and utilized the ggplot2 tool to show the results.

### Analysis of immune infiltration of hub gene in pan-cancer

Each tumor ImmuneScore and StromalScore were analyzed using the ESTIMATE R package, while correlations between the hub and these three scores were performed using the spearman algorithm. The relationship between *RBP1* and various immune cell types was analyzed in the TIMER2.0 database (timer.cistrome.org) using multiple algorithms such as TIMER, CIBERSORT, CIBERSORT-ABS, QUANTISEQ, XCELL, EPIC, and TIDE. To display the findings of this investigation, we utilized the “ggplot2” R package.

### Correlation analysis of hub gene and immune checkpoint gene

Each sample's gene expression data was collected for 36 Stimulatory Immune Checkpoints. Then, we identified the relationship between immune checkpoint genes and hub genes using the spearman method.

### Correlation analysis of hub gene with DNA methyltransferase, MMR gene, and cancer-related functional status

How four methyltransferases and hub genes were related was studied, DNMAT1, DNMT2, DNMT3A, and DNMT3B. And their expression levels correlated with five DNA mismatch repair (MMR) genes (MLH1, MSH2, MSH6, PMS2, and EpCAM). In addition, the correlation between *RBP1* and 14 cancer-related functional states in each tumor was analyzed and visualized using the “ggplot2” R package.

### Evaluation of *RBP1* mutation, TMB, and MSI in pan-cancer

To understand the mutation characteristics and location of *RBP1* in tumors, it was explored by using the CBioPortal database (https://www.cbioportal.org/). Also, for analysis, we employed Kaplan-Meier curves to understand the effect of mutations on patients' overall survival (OS). In addition, Spearman's test was used to analyze the relationship between *RBP1* expression and TMB and MSI of various tumors in the TCGA database. The “fast” R package visualized the results.

### Enrichment analysis

The correlation between *RBP1* and other genes was calculated in the GSE60681 dataset using the spearman algorithm, and the *P* value <0.05 was statistically significant. In addition, the top 500 similar genes associated with *RBP1* in 33 tumors were obtained from the GEPIA2 database. Genes related to *RBP1* in the GSE60681 dataset were intersected with the top 500 similar genes in 33 tumors. The Gene Ontology (GO) project was conducted in 2006 using the R package “clusterprofiler” to analyze intersecting genes (including molecular functions (MF), cellular components (CC), and biological processes (BP)). KEGG: Kyoto encyclopedia of genes and genomes. We used this analysis to predict hub genes' potential molecular bodily functions and signaling pathways. All tumor samples were split into high- and low-expression groups in various malignancies based on the median *RBP1* expression level. The function and mechanism of *RBP1* were investigated using gene set enrichment analysis (GSEA).

### Drug sensitivity analysis

To understand the potential drugs targeting *RBP1*, we analyzed the relationship between *RBP1* expression and drug sensitivity using the CallMiner database (http://discover.nci.nih.gov/cellminer/).

## Results

### GSVA

**Figure 1:**
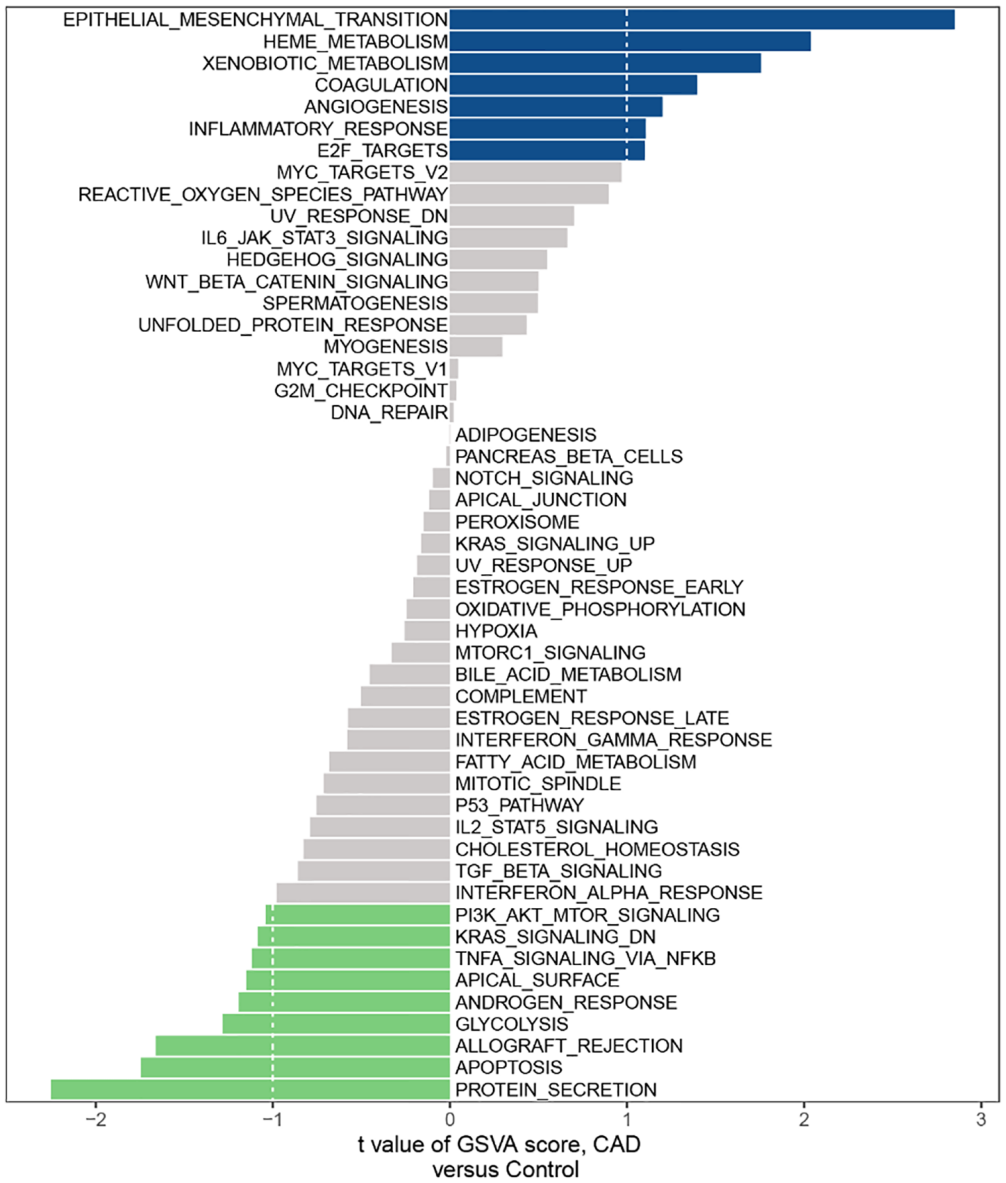
Hallmark gene set related to CAD in the GSE60681 dataset.

According to the GSVA results, we found a few mechanisms involved in immunity affected in CAD, such as IL-6/JAK/STAT3 SIGNALING, INFLAMMATORY RESPONSE, TNFA SIGNALING VIA NFKB, PI3K AKT MTOR SIGNALING and IL2 STAT5 SIGNALING (Figure 1).

### Co-expression network analysis of CAD

**Figure 2:**
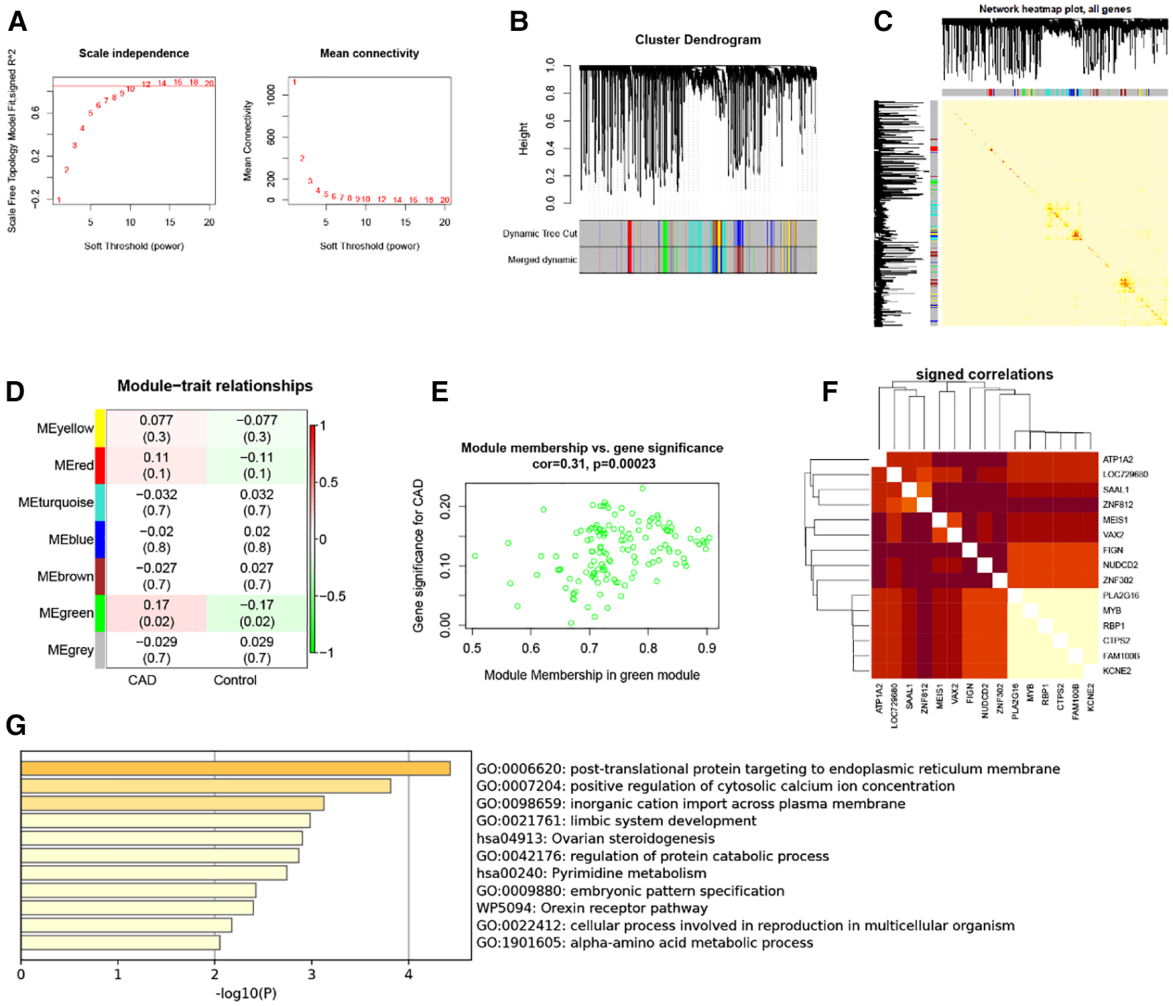
WGCNA. (**A**) 1–20 soft threshold power analysis using scale-free fit index (left) and analysis of mean connectivity (right). (**B**) Gene categorization using hierarchical clustering, with various hues signifying various modules. (**C**) The heatmap of the total gene network; (**D**) Heat map showing module features associated with CAD and Control group. (**E**) Gene scatterplot in the green module. (**F**) Correlations between 15 genes. (**G**) GO and KEGG analysis in green module gene.

Five thousand genes with differential expression were selected for further study. When using 0.85 as the correlation coefficient threshold, we chose a soft threshold power of 14 (Figure 2A). We set MEDissThres to 0.2 based on WGCNA analysis to combine related modules and generated seven modules (Figure 2B), including yellow, red, turquoise, blue, brown, green, and grey modules. Unable to be included in any module, genes were placed in the gray module. This gray module was not included in the further analysis. In addition, these modules were independent of other modules (Figure 2C). Analysis of the module-trait relationships revealed that the green module had the most significant relationship with CAD (Figure 2D). The relevance of these genes in the green module for CAD is depicted in Figure 2E. Note that: when GS >0.15, MM value >0.8, 15 genes in the green module (*KCNE2, FAM100B, CTPS2, RBP1, MYB, PLA2G16, ZNF302, NUDCD2, FIGN, VAX2, MEIS1, ZNF812, SAAL1 LOC729680* and *ATP1A2*) were of high significance for CAD (Figure 2F). Therefore, these genes can be considered candidate hub genes. Enrichment analysis of the green module showed that these module genes are involved in functions and pathways such as post-translational protein targeting to endoplasmic reticulum membrane, positive regulation of cytosolic calcium ion concentration, and inorganic cation import across the plasma membrane (Figure 2G).

### Results of the identification and expression level analysis of immune-related hub genes

**Figure 3:**
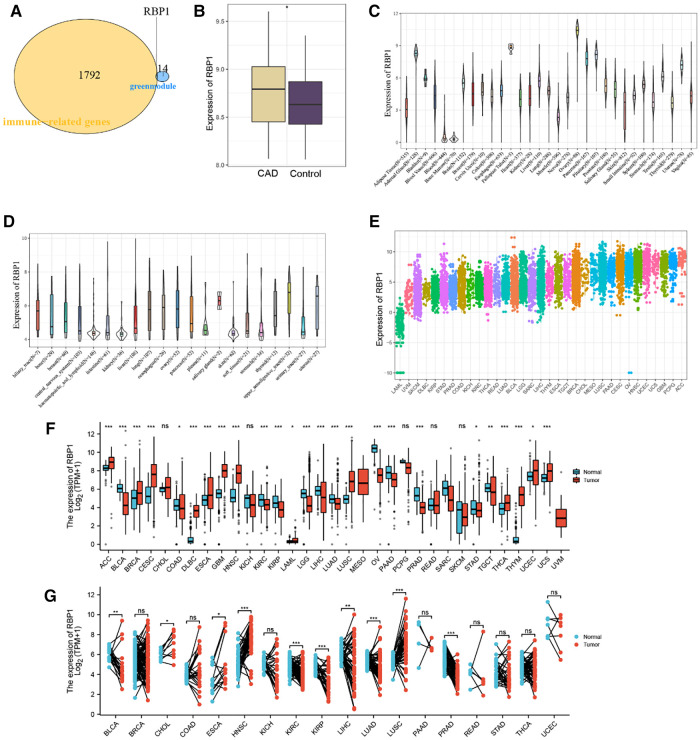
Identification and expression level analysis of immune-related hub genes. (**A**) Venn diagram showing *RBP1* as an intersection gene of immune-related genes and green module. (**B**) Expression level of *RBP1* in CAD and control. (**C**) *RBP1* expression levels in healthy tissues are listed in the GTEx database. (**D**) Tumor cell lines in the CCLE database's *RBP1* expression levels. (**E**) *RBP1* expression in tumor tissues, according to the TCGA database. (**F**) The merging of data from the GTEx database and TCGA for the tumor tissues has shown the *RBP1* expression levels in tumor tissues and normal tissues. (**G**) *RBP1* expression levels in TCGA database paired with normal and tumor tissues expression levels.

RBP1 was found to be an immune-related hub gene after 1,793 immune-related genes from the import database were retrieved and intersections with 15 crucial genes from the green module were considered (Figure 3A). The results showed that RBP1 was markedly overexpressed in CAD based on data from the GSE60681 dataset (Figure 3B). *RBP1* expression was highest in the Ovary, Fallopian Tube, and Adrenal Gland, and lowest in blood, bone marrow, and muscle, according to the analysis of standard tissue samples from the GTEx database (Figure 3C). The CCLE database's details on tumor cell lines showed (Figure 3D) that the *RBP1* was more uniform in different tumor cell lines. Data on tumor tissue samples from the TCGA database showed that *RBP1* expression was highest in breast invasive carcinoma (BRCA), lung squamous cell carcinoma (LUSC) and lowest in lung squamous cell carcinoma (LAML), uveal Melanoma (UVM) (Figure 3E). After merging standard tissue samples from the GTEx database and tumor tissue samples from TCGA (Figure 3F). We found that *RBP1* was highly expressed in adrenocortical carcinoma (ACC), ovarian serous cystadenocarcinoma (OV), colon adenocarcinoma (COAD), lymphoid neoplasm diffuse large B-cell lymphoma (DLBC), esophageal carcinoma (ESCA) and glioblastoma multiforme (GBM). Paired average and tumor tissue data from the TCGA database showed low expression of *RBP1* in Bladder Urothelial Carcinoma (BLCA), cholangiocarcinoma (CHOL), esophageal carcinoma (ESCA), head and Neck squamous cell carcinoma (HNSC), kidney renal clear cell carcinoma (KIRC), and prostate adenocarcinoma (PRAD) (Figure 3G).

### Survival prognosis analysis of *RBP1* in cancers

**Figure 4:**
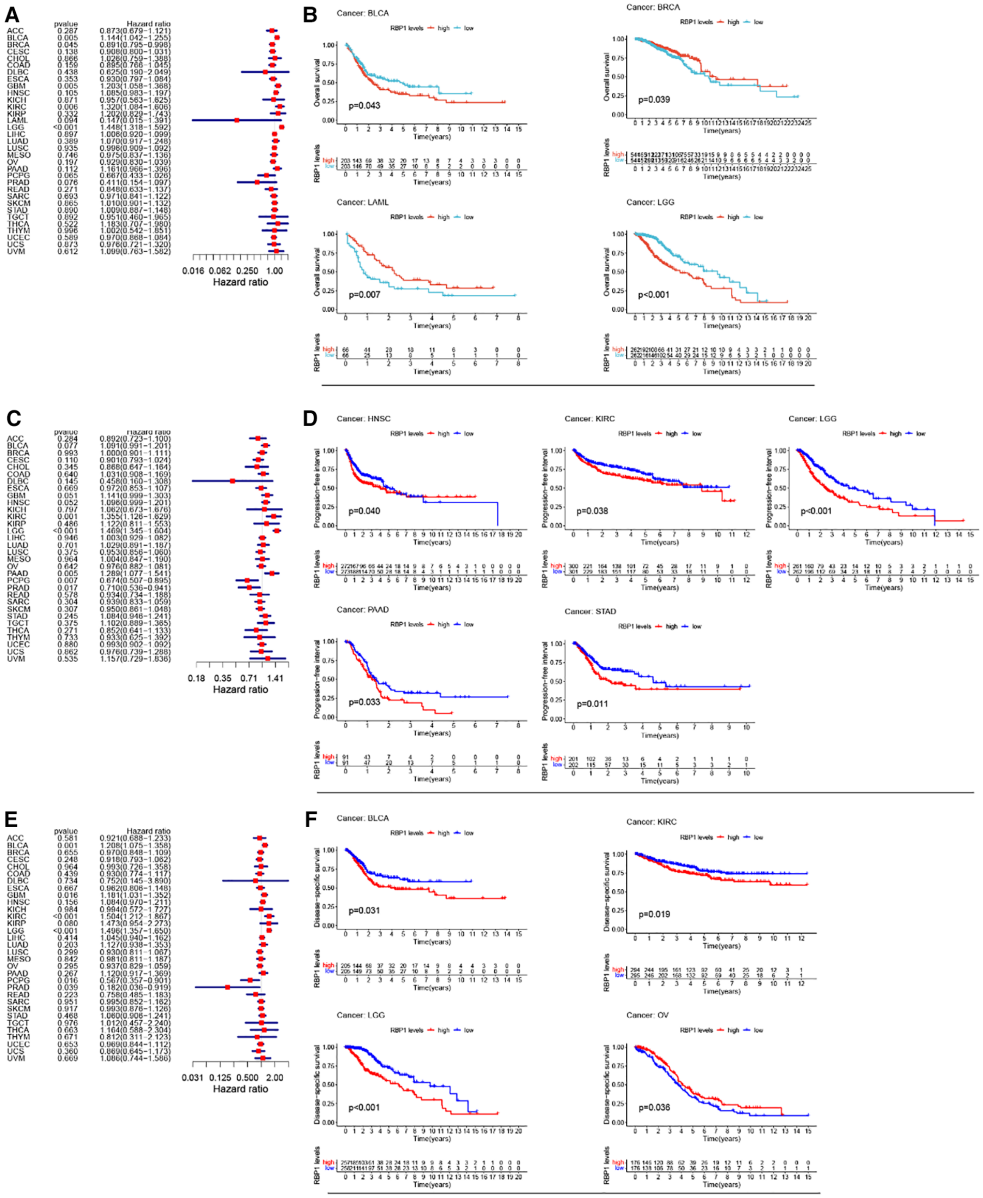
*RBP1*'s impact on cancer patients’ prognoses. (**A**) One-way cox analysis of *RBP1* in OS. (**B**) KM analysis of *RBP1* in OS. (**C**) One-way cox analysis of *RBP1* in PFI. (**D**) KM analysis of *RBP1* in PFI. (**E**) One-way cox analysis of *RBP1* in DSS. (**F**) KM analysis of *RBP1* in DSS.

Univariate cox analysis of OS showed (Figure 4A) that *RBP1* was a protective factor for BRCA and a risk factor for brain lower grade glioma (LGG), BLCA, GBM, and KIRC. KM analysis of OS showed (Figure 4B) that high expression of *RBP1* improved the prognosis of patients with BRCA and LAML and reduced survival in patients with BLCA and LGG. Univariate cox analysis of PFI showed (Figure 4C) that *RBP1* was a risk factor for patients with pancreatic adenocarcinoma (PAAD), KIRC, LGG and a protective factor for patients with pheochromocytoma and paraganglioma (PCPG) and PRAD. KM analysis of PFI showed (Figure 4D) that low expression of *RBP1* prolonged survival in patients with stomach adenocarcinoma (STAD), HNSC, KIRC and PAAD. Univariate cox analysis of DSS showed (Figure 4E) that *RBP1* was a risk factor for BLCA, LGG and a protective factor for PCPG and PRAD. KM analysis of the DSS showed (Figure 4F) that low expression of *RBP1* predicted a better prognosis for patients with BLCA and LGG and was a danger signal for survival in patients with OV.

### Methylation analysis and clinical staging results

**Figure 5:**
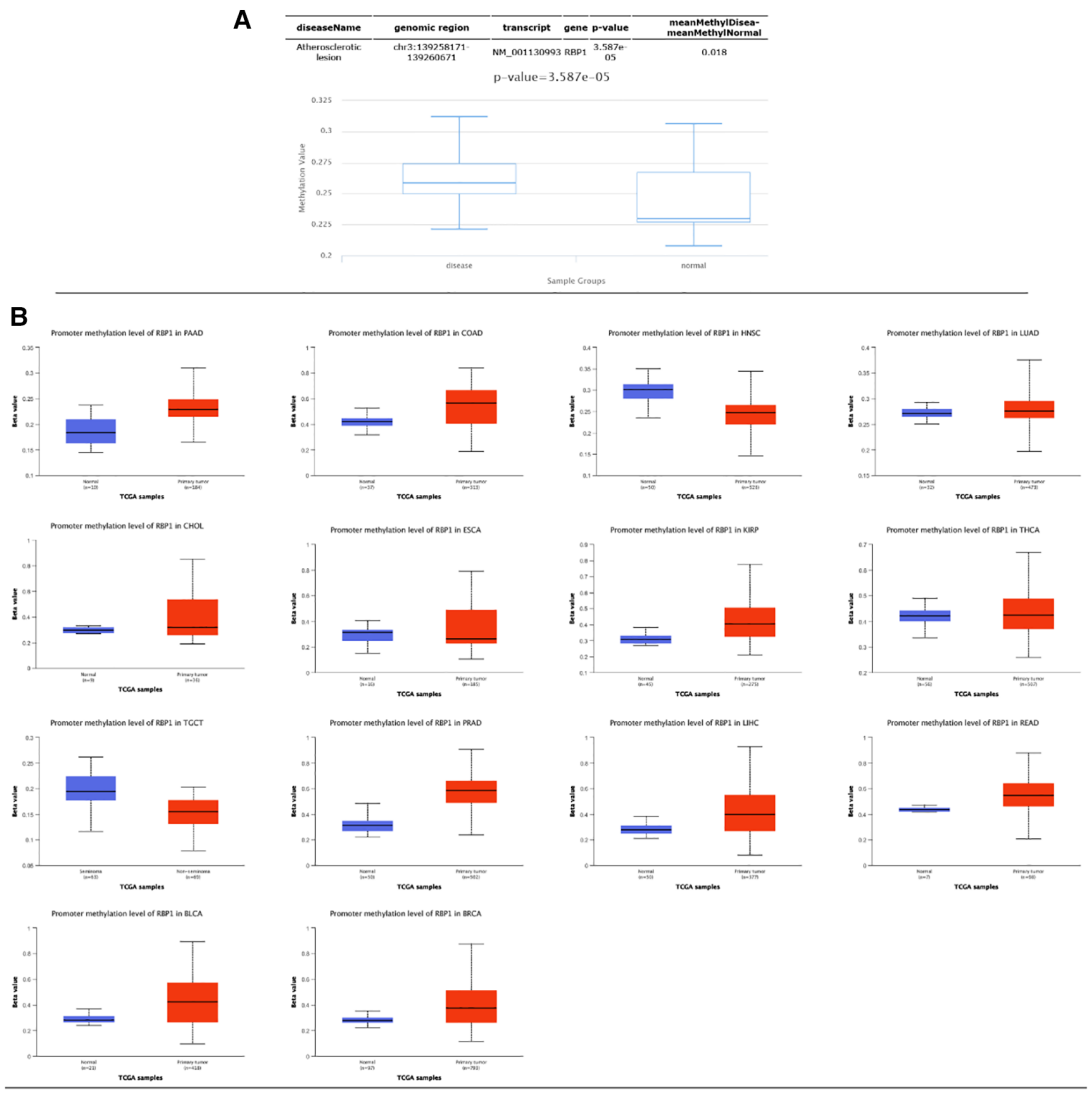
Methylation and clinical staging analysis. **(A)** Significant hypermethylation of *RBP1* in atherosclerosis. (**B**) Methylation analysis of *RBP1* in individual tumors, of which it was significant in 14 tumors.

**Figure 6:**
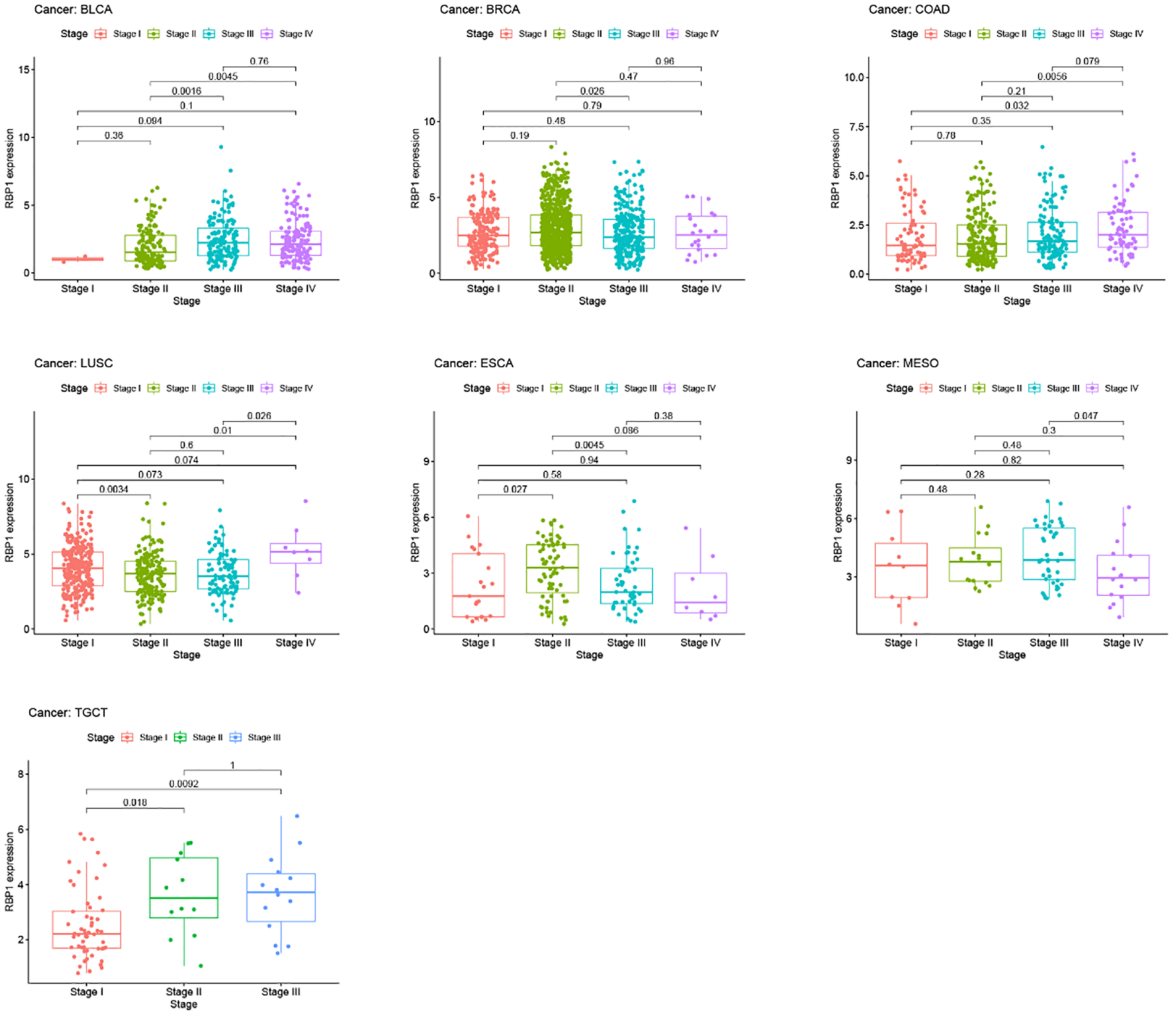
Clinical staging analysis of *RBP1* in tumors.

Results from the disease myth 3.0 database showed (Figure 5A) that *RBP1* was significantly hypermethylated in CAD. Based on the results of the ualcan database (Figure 5B), we found that *RBP1* was particularly hypermethylated in PAAD, COAD and BRCA. *RBP1* was significantly hypermethylated in testicular germ cell tumors (TGCT), HNSC and ESCA. The clinical staging results showed (Figure 6) that *RBP1* expression was higher in higher stages of BLCA, COAD and TGCT and lowered in higher stages of mesothelioma (MESO), BRCA and ESCA.

### Immuno-infiltration analysis of CAD

**Figure 7:**
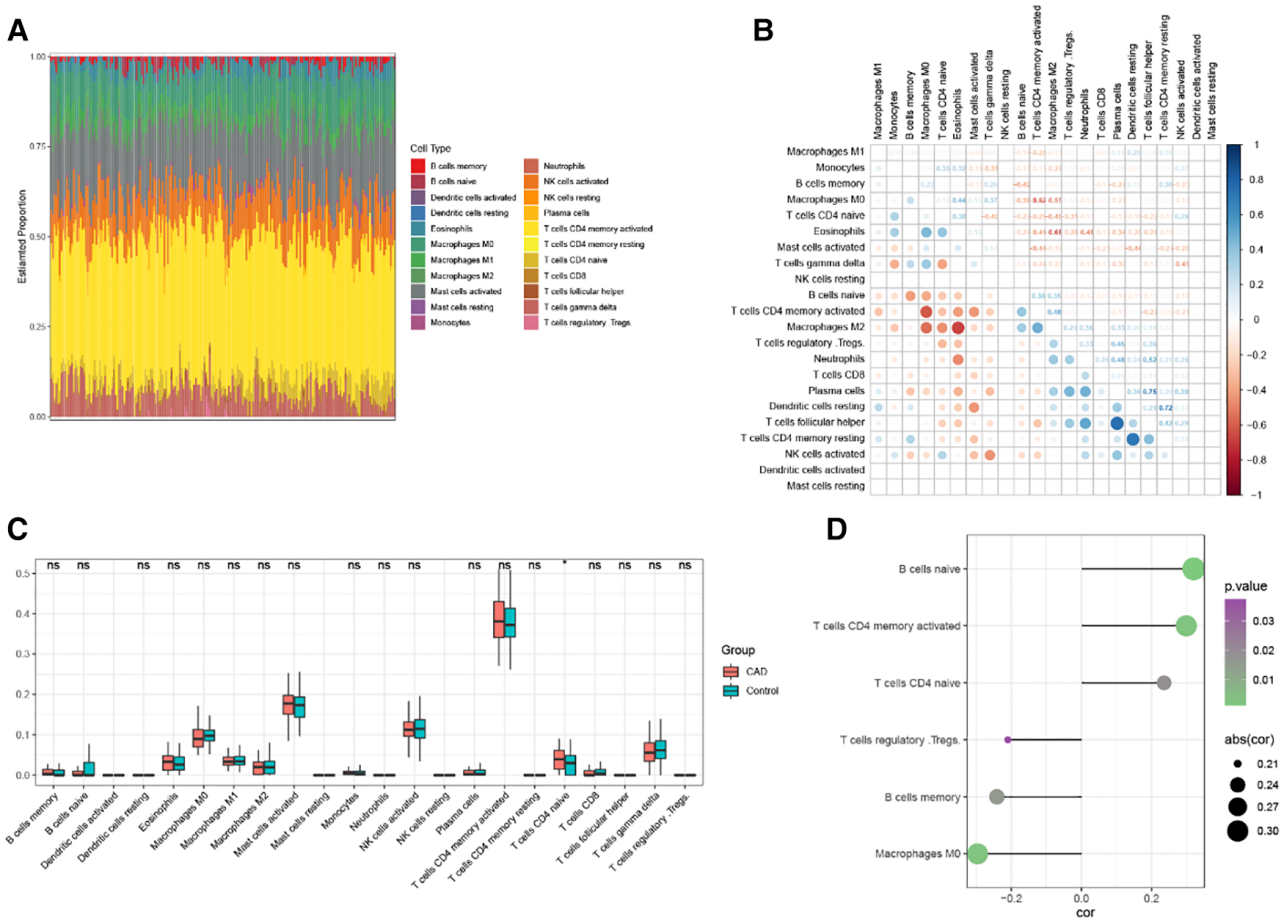
Analysis of CAD immune infiltration. (**A**) Each sample's 22 immunological cells as a percentage. (**B**) Correlation analysis of 22 immune cells. (**C**) 22 immune cells’ expression levels differ between CAD and Control. (**D**) Correlation analysis of *RBP1* and immune cells.

Using the cibersort technique, we analyzed the connection between the CAD phenotype and immune cell infiltration. The box plot shows the proportional proportions of immune cell subgroups (Figure 7A). The analysis indicated that T cells with CD4 memory activation, CD4 naive activation, and activated Mast cells accounted for a more significant amount. Figure 7B displays the correlation heat map of 22 immune cells. Violin plots of immune cell infiltration differentials showed that T cells CD4 naïve significantly differed in CAD (Figure 7C). *RBP1* was considerably positively connected with B cells naive, T cells CD4 memory activated, T cells CD4 naive, and negatively correlated with Macrophages M0, B cells memory, and T cells regulatory (Tregs), according to the correlation between *RBP1* and immune cells (Figure 7D).

### Immune infiltration analysis of pancytopenia

**Figure 8:**
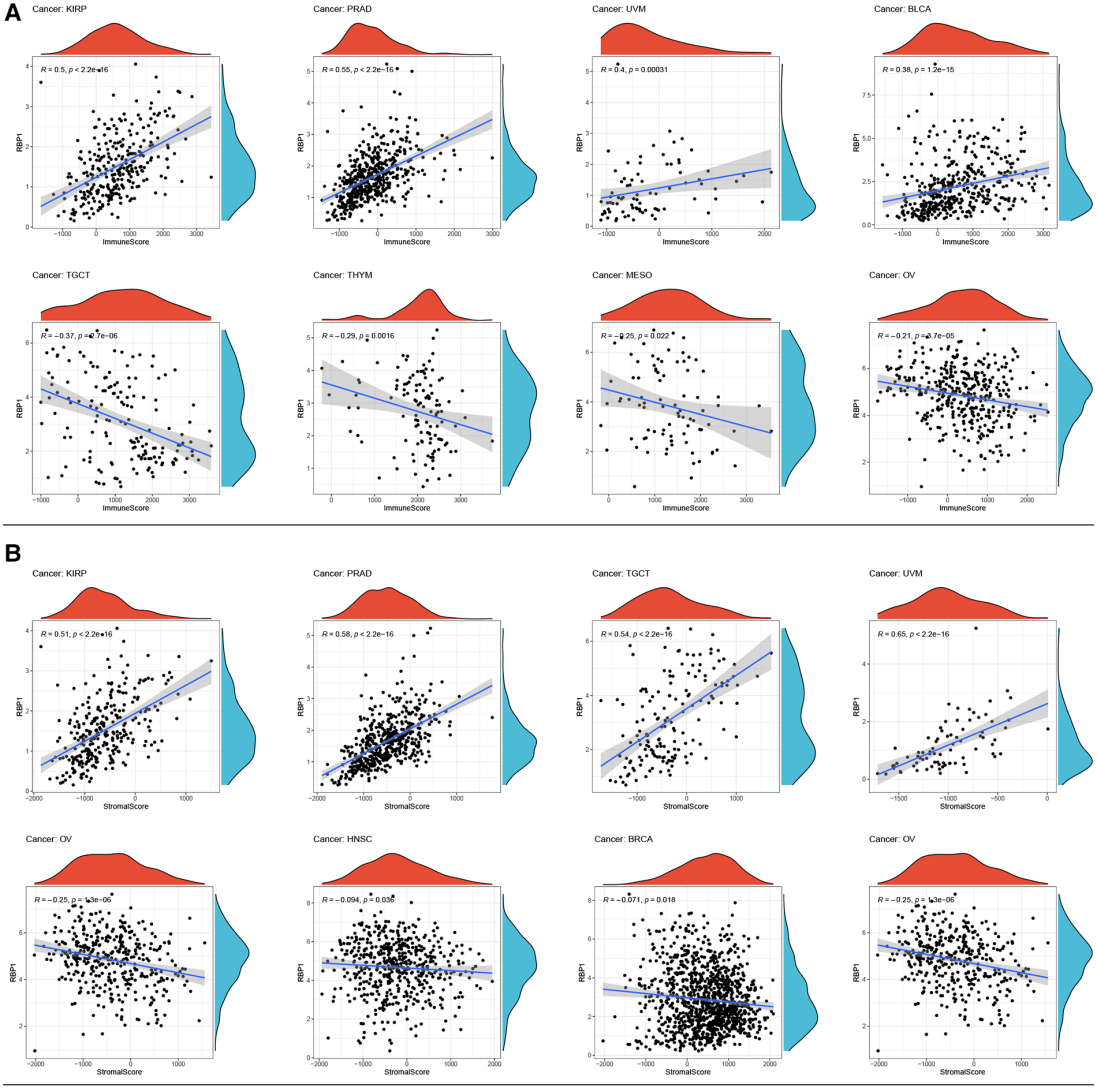
Analysis of *RBP1*'s relationships with ImmuneScore and StromalScore. **(A)** Results of correlation analysis of *RBP1* with ImmuneScore. (**B**) Correlation analysis of *RBP1* with StromalScore.

**Figure 9:**
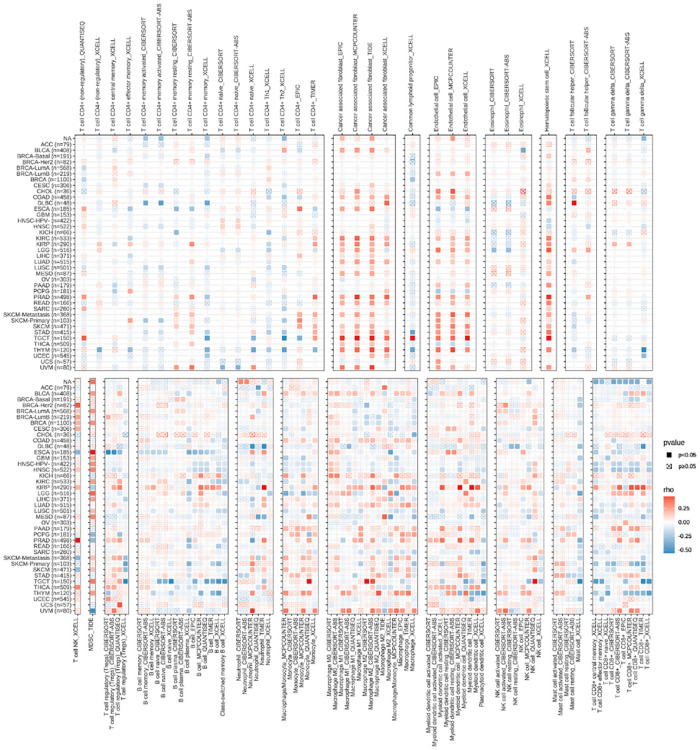
The relationships between *RBP1* expression and the amount of immune cell infiltration in pan-cancer.

Top 4 positive and top4 negative correlation results of *RBP1* were displayed with ImmuneScore and StromalScore. Figure 8A showed that *RBP1* was significantly positively associated with the ImmuneScore of PRAD, and UVM and negatively associated with the ImmuneScore of TGCT, MESO and OV. Regarding StromalScore (Figure 8B), *RBP1* had a strong negative relationship with the StromalScore of OV and a significant positive connection with the StromalScore of PRAD, TGCT, and UVM. The correlation heat map revealed that *RBP1* was highly connected with various immune cells in pan-cancer, including CD8+ T cells, neutrophils, macrophages, cancer-associated fibroblast (CAF), endothelial cells, and hematopoietic stem cells (Figure 9).

### Correlation analysis of hub gene and immune checkpoint gene

**Figure 10:**
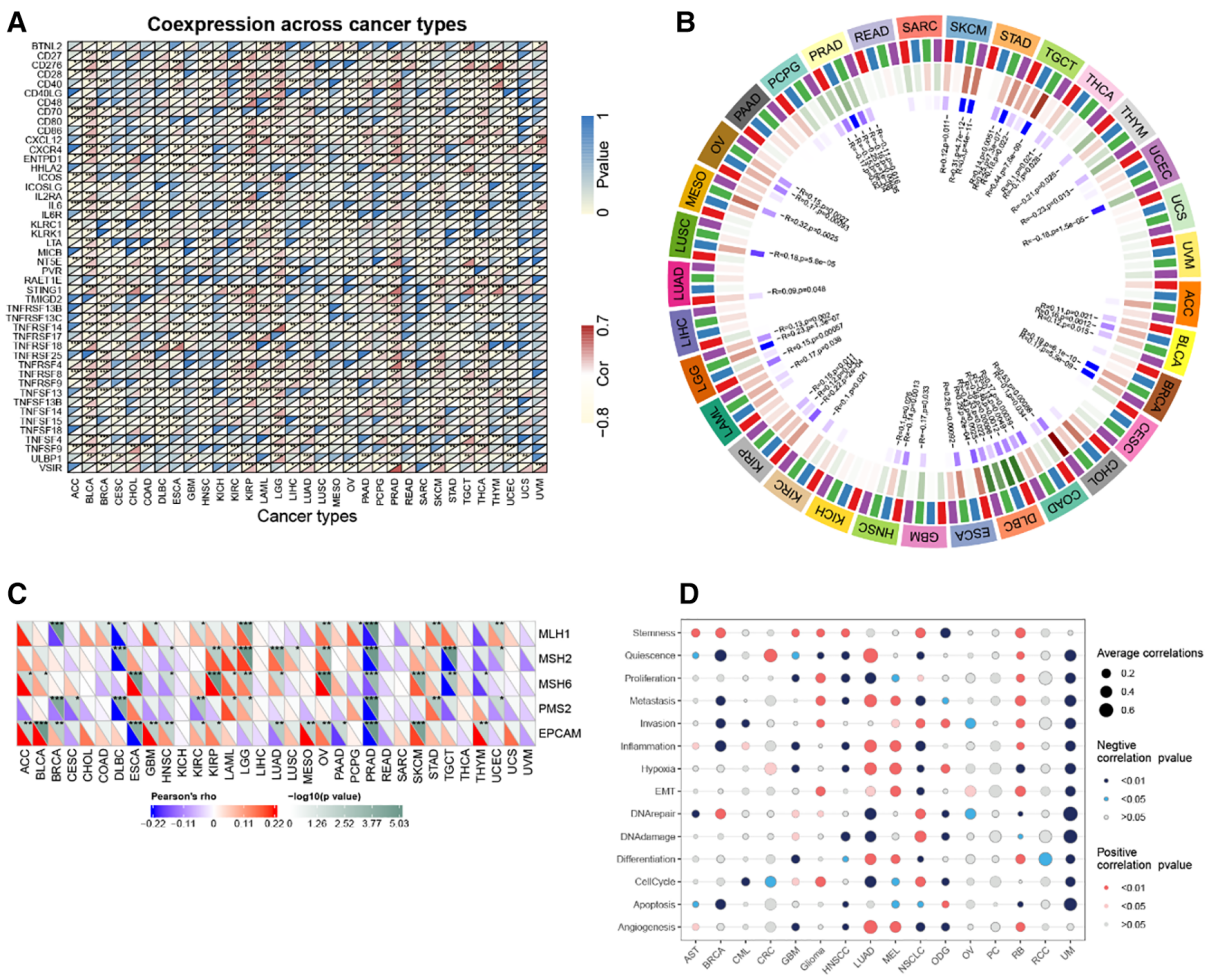
Correlation analysis. (**A**) *RBP1* expression and immune checkpoint gene correlation analyses. (**B**) DNA methyltransferases and *RBP1* expression correlation analysis. (**C**) *RBP1* expression and MMR gene correlation analysis. (**D**) The functional status of cancer and *RBP1* expression.

In the majority of tumors, *RBP1* was discovered to have a substantial and positive correlation with immune checkpoint genes such as CD27, CD276, CD28, CD40, CD86, CXCL12, CXCR4, ENTPD1, IL2RE, IL6, IL6R, MICB, PVR, STING1, TNFRSF14, TNFRSF17, TNFRSF25, TNFSF13, TNFSF13B, TNFSF14, TNF (Figure 10A).

### Analysis of hub gene and DNA methyltransferase, MMR gene and cancer-related functional status

The relationships between RBP1, the four DNA methyltransferases, and each other was investigated. The results showed that RBP1 expression was significantly correlated with at least one DNA methyltransferase in cancers other than PAAD, UVM, and ACC (Figure 10B). In addition, how *RBP1* related to the degree of MMR gene mutations was studied. The results showed a strong association between *RBP1* expression in PRAD and five MMR genes' levels of transformation (Figure 10C). Finally, *RBP1* was significantly and negatively correlated with 12 different cancer-related functional states in UM, respectively (Figure 10D).

### Analysis of *RBP1* correlation with mutation, TMB, MSI

**Figure 11:**
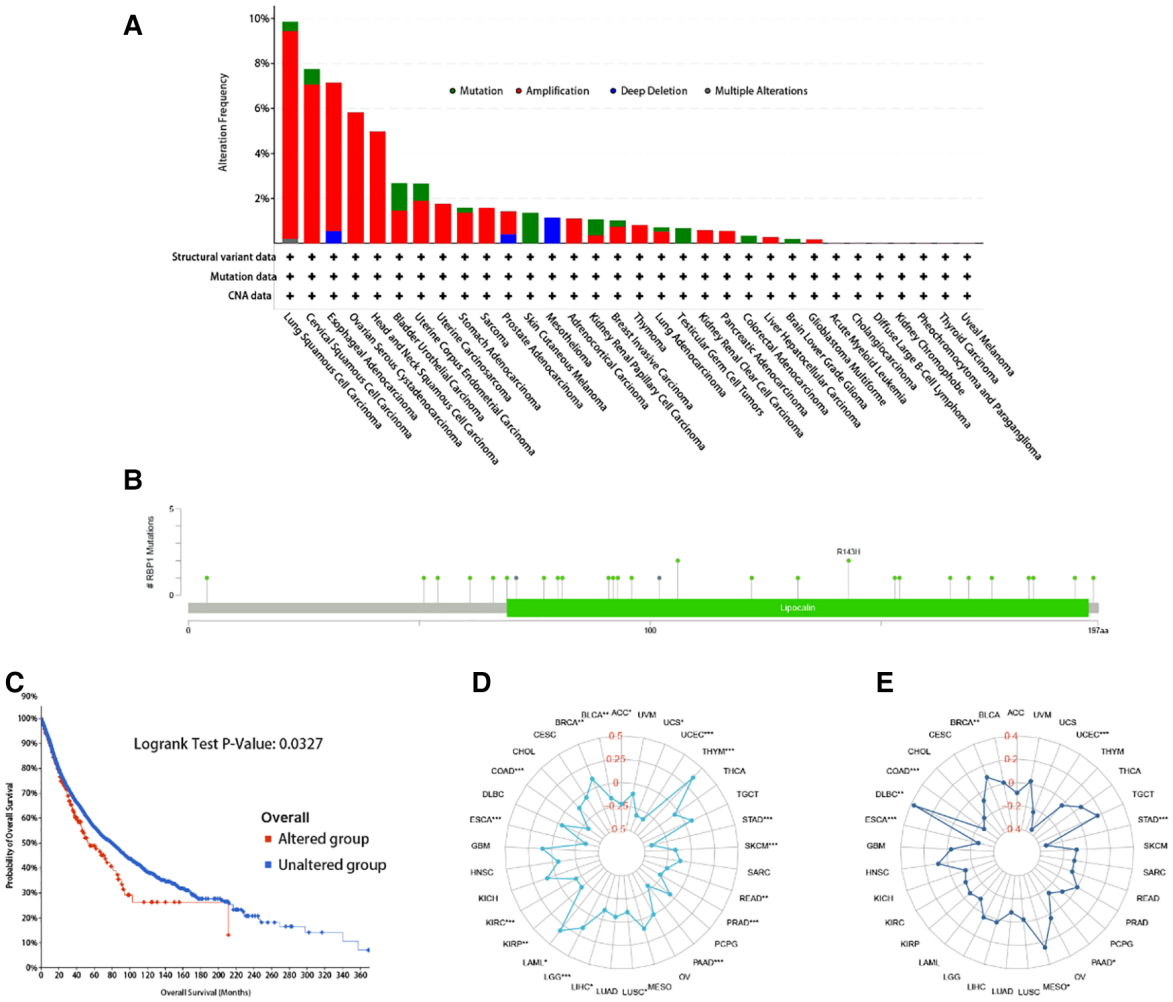
The *RBP1* mutation signature landscape in TCGA. Each mutation type's (**A**) and mutation site's proportional frequency (**B**). (**C**) Kaplan–Meier plots showing the comparison of OS in cases with/without *RBP1* gene alterations in Overall developed using the cBioPortal tool. The radargram of TMB (**D**), MSI (**E**), and *RBP1* expressions in various cancers.

*RBP1* mutation type was dominated by Amplification. The frequency of *RBP1* mutations was highest in lung squamous cell carcinoma (Figure 11A). The *RBP1* mutation sites are shown in Figure 11B. The Kaplan-Meier analysis's findings revealed that patients with malignancies in the altered group had a worse prognosis (Figure 11C). Figure 11D demonstrates that *RBP1* is significantly associated positively with TMB in the BRCA, LGG, and LAML. Additionally, it had a strong negative correlation with TMB in the following: BLCA, ACC, STAD and COAD. *RBP1* demonstrated a statistically significant positive connection with MSI in BRCA, MESO, and DLBC, as illustrated in Figure 11E. Additionally, it had a markedly negative relationship with MSI in the STAD, PAAD, ESCA, and COAD.

### Similarity genes of *RBP1* and GO and KEGG analysis

**Figure 12:**
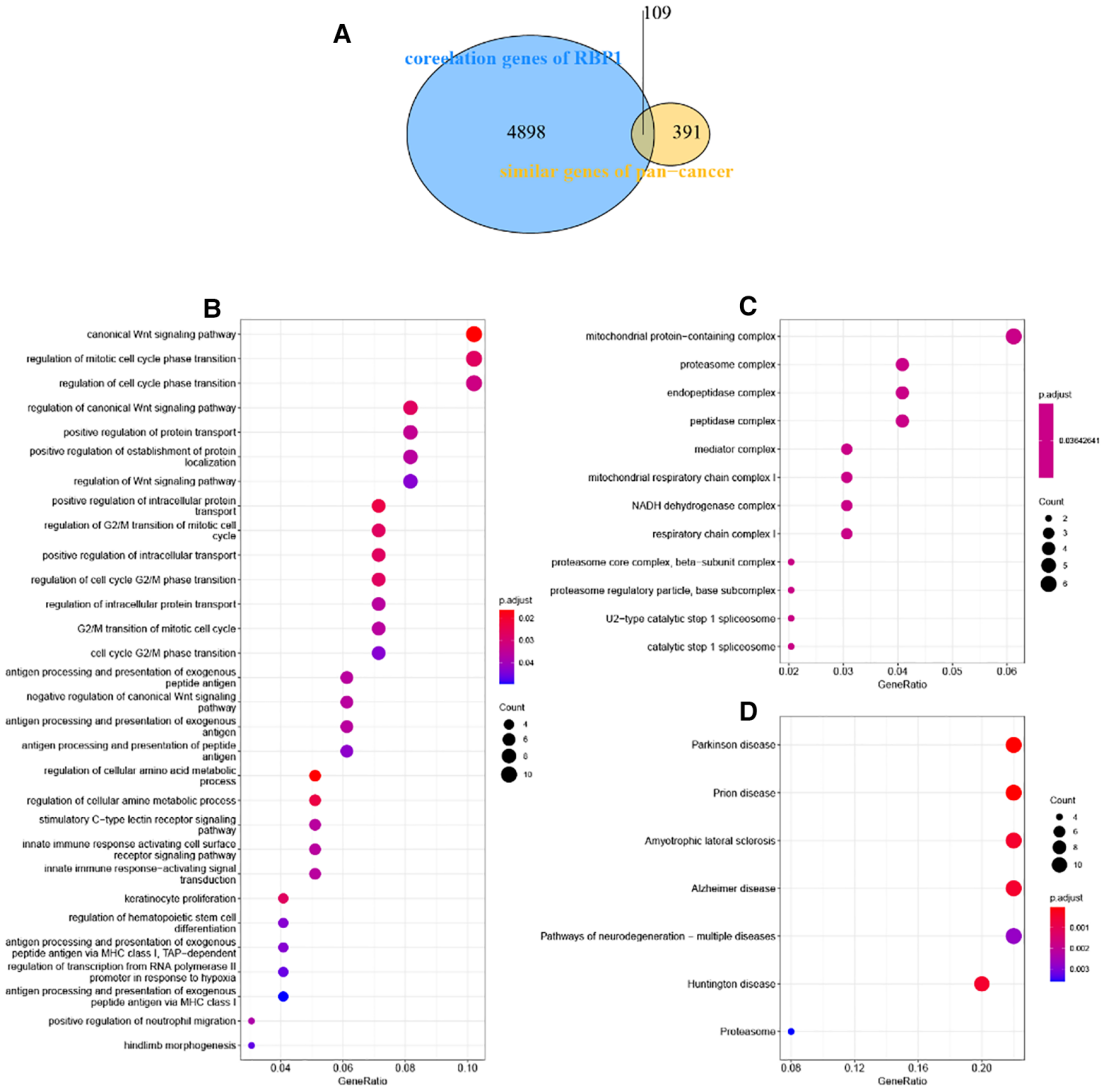
Enrichment analysis. (**A**) Venn diagram showing 109 similarly expressed pattern genes. (**B**) Biological process. (**C**) Molecular function. (**D**) KEGG.

**Figure 13:**
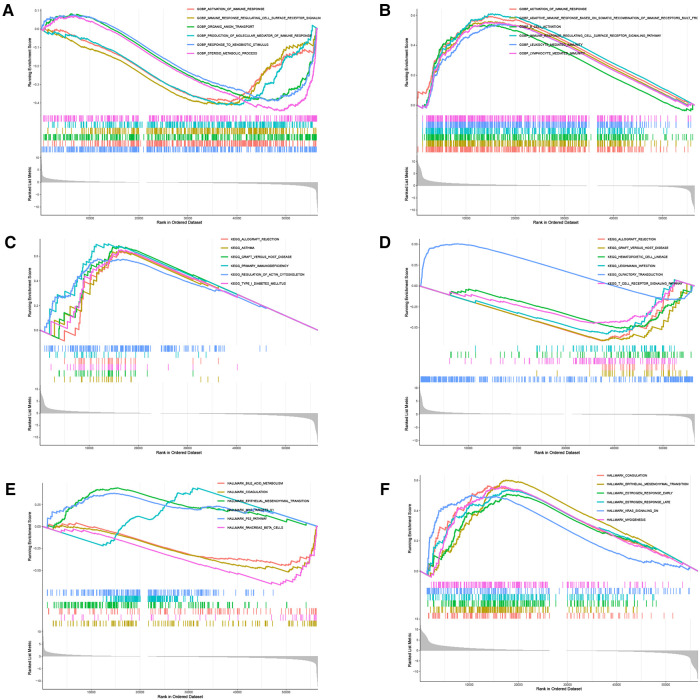
GSEA. Analysis of *RBP1* enrichment in BP (**A–B**), KEGG(**C–D**), and Hallmark (**E–F**).

There were 109 intersections of *RBP1* with similar expression pattern genes between dataset GSE60681 and pan-cancer (Figure 12A). The GO and KEGG analysis results of *RBP1* are shown in Fig. In BP, it was significantly enriched in the canonical wnt signaling pathway, regulation of mitotic cell cycle phase transition, regulation of cell cycle phase transition, regulation of canonical wnt signaling pathway, etc. (Figure 12B). In CC, *RBP1* is associated with mitochondrial protein-containing complex, proteasome complex, and endopeptidase complex. It was significantly enriched in peptidase complex (Figure 12C). We have no enrichment found in MF. In KEGG, they were significantly increased in Parkinson's disease, prion disease, amyotrophic lateral sclerosis, Alzheimer's disease, and other signaling pathways (Figure 12D). We also used GSEA to investigate the function and mechanism of *RBP1* in various gene sets. Our results showed that *RBP1* was connected to the ACTIVATION OF IMMUNE RESPONSE and IMMUNE RESPONSE REGULATING CELL SURFACE RECEPTOR SIGNALING PATHWAY in BP (Figures 13A,B). In KEGG, *RBP1* was linked to GRAFT VERSUS HOST DISEASE and ALLOGRAFT REJECTION (Figures 13C,D). In HALLMARK, *RBP1* was associated with activation or inhibition of COAGULATION and EPITHELIAL MESENCHYMAL TRANSITION (Figures 13E,F).

### Sensitivity analysis of *RBP1* and drugs

**Figure 14:**
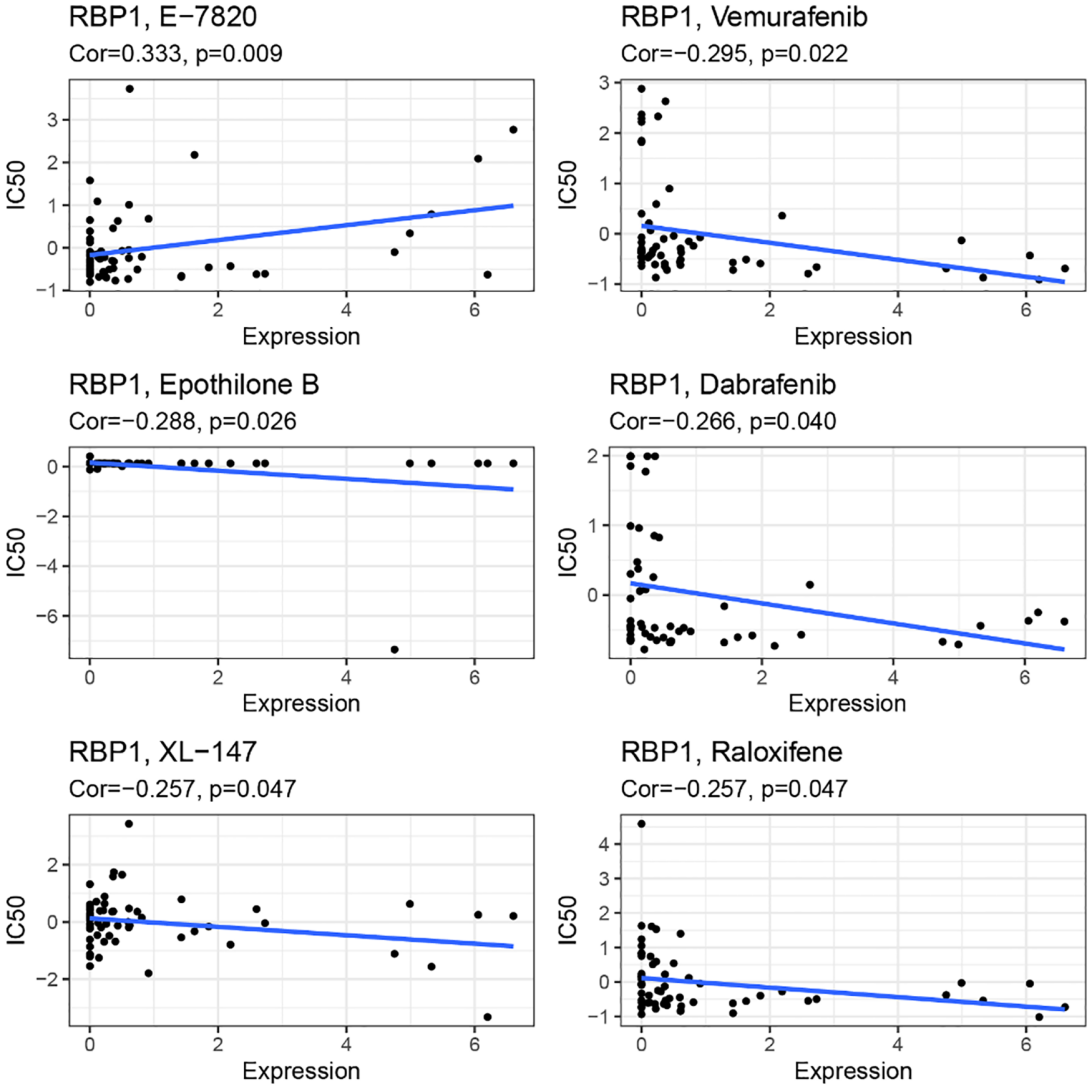
Drug sensitivity analysis.

*RBP1* expression and E-7820 have a highly significant positive correlation in the CallMiner database. And it was significantly negatively associated with five drugs, including vemurafenib, epothilone, dabrafenib, XL-147, and raloxifene (Figure 14).

## Discussion

To date, CAD and cancer are the two major diseases that threaten human health and lead to death. As the world ages, the number of cancer survivors increases, but many cancer treatments and drugs are cardiotoxic. For example, combining bleomycin, bevacizumab, and vincristine can increase the CAD risk by 1.5–7 times (19). Therefore, cancer patients often have coexisting heart disease (20) and are most often seen with CAD in conjunction with cancer (21, 22). On the other hand, immune cells and responses are a crucial link in the onset of cancer and CAD, and immunotherapy has been widely used in treating cancer. Still, the connection between immunity in CAD and cancer has rarely been reported. A recent study has shown that some genes are strongly linked to CD8+ T immune cells responsible for the onset of CAD and cancer (23) and may provide new therapeutic targets for CAD and coronary artery disease. However, the precise mechanisms are not yet known.

In this research, WGCNA was deployed to identify modules significantly associated with CAD in the GSE60681 dataset, and among them, the most immunologically relevant gene, *RBP1*, was found. *RBP1* affects retinoic acid metabolism by reducing retinol transport, is involved in various physiological functions, and plays a crucial part in cancer genesis (24, 25). *RBP1* expression levels are highly expressed in CAD and vary in different cancers. *RBP1* was strongly expressed in 25 cancers containing ACC, BLCA, BRCA, etc. We paired standard tissue samples from the GTEx database with tumor tissue samples from the TCGA. After that, *RBP1* was lowly expressed in 10 tissues, including BLCA, CHOL, ESCA, etc. Kaplan-Meier survival analysis and univariate cox analysis revealed that *RBP1*'s expression level in malignant tumors was linked to poor prognosis. *RBP1* silencing is a common epigenetic phenomenon in many human cancers (26). Low or defective *RBP1* gene expression is closely associated with developing breast cancer, kidney cancer, prostate cancer, lung cancer, and endometrial cancer (27–29). The prognosis is worse when *RBP1* is expressed more frequently. It is closely associated with the malignant biological processes of self-renewal, invasion, and migration in non-glioblastoma diffuse glioma (non-GDG). The NF-*κ*B signaling pathway is related to *RBP1*-dependent malignancy in non-GDG (30). *RBP1* is highly expressed in lung adenocarcinoma and mediates cell proliferation and differentiation by up-regulating Akt/Erk/ EGFR pathway, which is associated with increased tumor grade (31). The expression of *RBP1* in tongue squamous carcinoma cells (TSCC) correlates with the degree of tumor differentiation and lymph node metastasis (32), and *RBP1* overexpression in oral squamous cell carcinoma (OSCC) promotes the development of cellular autophagy and facilitates the growth and invasion of OSCC (25). Consistent with our results, these studies confirmed the close relationship between *RBP1* and cancer and prognosis. According to the findings of our clinical staging, *RBP1* expression was higher in the intermediate and late stages of four malignancies, including BLCA and COAD, but was lower in the higher stages of BRCA, ESCA, and MESO tumors. Increased expression of *RBP1* may reflect the invasion of cancer. It may be possible to monitor the expression level of *RBP1* to evaluate the development of CAD patients with cancer.

The body's immune system regulates tumor occurrence and development. At the same time, macrophages and smooth muscle cells, under the synergistic effect of immune cells and inflammatory factors, phagocytosis of lipids to form foam cells, which promotes the formation of atherosclerosis, which is the mechanism of CAD occurrence and development (33). Interleukin (IL)-1β can reduce the risk of CVD in patients with residual inflammation (34), and methotrexate has anti-inflammatory effects. Although it can treat CVD, it cannot reduce risk and mortality (35). CD4+ T cell-mediated immune responses are involved in the development of CAD (36, 37) and affect the stability of atherosclerotic plaques through the secretion of interferon (INF-*γ*) in Th1 cells, leading to acute coronary syndrome (ACS) (38, 39). On the other hand, CD8+ T cells are the main cytokine secreting INF-*γ*. In tumor immunity, INF-*γ* enhances the inflammatory response associated with Th17 cells promoting tumor development (40), and in the tumor microenvironment, INF-*γ* inhibits tumor-specific evasion of immune surveillance (41). We analyzed immune cell infiltration in CAD and cancer patients, and the analysis of immune infiltration in CAD showed a significant difference in T cells CD4 naïve in CAD and a significant positive correlation between *RBP1* and immune correlation with T cells CD4 naïve. The TIMER study revealed a substantial correlation between *RBP1* expression and the immune infiltration of malignancies. CD8+ T cells, CD4+ T cells, cancer-associated fibroblast, etc., were all strongly positively correlated with *RBP1* in various tumors. We concluded that *RBP1* might be a gene that influences the immune response involved in CAD and cancer development. We also calculated the relation between *RBP1* expression levels and immune scores to explore the link between *RBP1* and the tumor immune microenvironment. The findings demonstrated that PRAD and UVM tumor immune cell infiltration were positively connected with *RBP1*, and OV, HNSC, and BRCA tumor immune cell infiltration significantly negatively correlated with *RBP1*. According to this analysis, *RBP1* may impact the tumor's immune microenvironment and the course of cancer.

In addition to immune infiltration, gene mutations are also associated with developing malignant tumors. LGGs with isocitrate dehydrogenase (IDH) gene mutation are more likely to develop into secondary GBMs (42). FOXA1 gene mutation inhibits androgen signaling in prostate cancer and promotes tumor growth (43). We evaluated the mutation types of the *RBP1* gene in cancer and showed that *RBP1* is the predominant gene in the Amplification mutation type. In addition, according to Kaplan-Meier survival analyses, the set of tumors with *RBP1* mutations had a worse prognosis. TMB, MSI, and response to tumor immunotherapy are closely related (44, 45). In BLCA and ACC, *RBP1* expression had a negative relationship with TMB but was positively linked with TMB in BRCA, LGG, and LAML tumors. In BRCA, MESO, and DLBC, there was a substantial positive association between *RBP1* and MSI, whereas, in STAD, PAAD, and the other five tumors, there was a significant negative correlation. In addition, MMR variants can be used as a predictor of tumors (46), and mutations in the MMR gene constitute a considerable cause of MSI instability in patients with rectal cancer (CRC) (47). In previous studies, MMR deficiency, high TMB, and high MSI were shown to be more effective in immunotherapy (48). The expression levels of *RBP1* in PRAD were significantly and positively linked with the levels of mutation of five MMR genes, according to our further analysis of the relationship between *RBP1* and MMR gene mutation levels. *RBP1* was linked considerably with TMB, MSI, and MMR in various tumors, suggesting that *RBP1* could be used as a novel assay in clinical immunotherapy targeting specific populations.

Immunotherapy is one of the main methods to treat cancer. Immunotherapy mainly includes tumor vaccines, checkpoint inhibitors (ICBs), adoptive cell transfer therapy, etc. CAR-T therapy is the most widely used immunotherapy method, and its most common therapeutic targets are extracellular glycoproteins CD19, CD20, and B-cell surface antigens such as BCMA (49). However, there's no appropriate immunotherapy for the medical therapy of CAD. And in some studies of immunotherapy for CAD: the Canakinumab Anti-Inflammatory Thrombosis Outcome Study (CANTOS) and the Cardiovascular Inflammation Reduction Trial (CIRT), the results showed that optimal immunotherapy for CVD can only be reached by identifying new drug therapeutic targets and blocking specific inflammatory pathways of atherosclerosis (50). Immune checkpoint inhibitors of tumors mainly inhibit programmed cell death protein (PD) 1 and cytotoxic T lymphocyte-associated protein (CTLA) 4 are currently the primary approach to tumor immunotherapy (51). In addition, CTLA4 drives or inhibits plaque inflammation as a critical protein in regulating atherosclerosis. Animal experiments demonstrated that CTLA4 binding to abatacept protein inhibits the interaction between CD28-CD80/CD86 to inhibit atherosclerosis (52). We also looked at the relationship between *RBP1* and genes associated with immunological checkpoints. We discovered that *RBP1* was positively connected with immune checkpoint activation genes in most cancers, indicating that the amount of *RBP1* expression was closely related to immune cell infiltration of tumor cells and immune cell function. Immune checkpoint therapy might be an effective treatment for CAD. Patients with CAD combined with cancer have a hypercoagulable state of blood and coagulation disorders due to the tumor itself, which can exacerbate the progression of the disease (53). In addition, since drugs for cancer treatment are often accompanied by cardiac toxicity, fatal fulminant myocarditis caused by immune checkpoint inhibitors has also been reported in the literature, even if the mechanism of immune checkpoint inhibitors is different from that of previous chemotherapy (54). Considering that *RBP1* may be a potential gene related to CAD and cancer, we examined how *RBP1* and anticancer drug sensitivity are related using the CallMiner database. The outcomes demonstrated a positive connection between the IC50 of E-7820 and the expression of *RBP1*. However, it was negatively correlated with the IC50 of Vemurafenib, Epothilone B, Dabrafenib, XL−147, and Raloxifene. This result further expands the clinical value of *RBP1*.

The mechanism and function of *RBP1* are further explored. Analysis of enrichment showed that *RBP1* was significantly enriched in biological processes such as mitochondrial protein-containing complex mitochondrial, proteasome complex, respiratory chain complex I, and cell mitotic cycle. The mitochondria play an essential role in cancer development; mitochondrial ubiquinol oxidation in tumor cells is required for cancer proliferation (55). Pten-induced kinase 1 (PINK1)/ Parkin-mediated mitochondrial autophagy drives the proliferation of vascular smooth muscle cells (VSMCs), which in turn leads to atherosclerotic lesions and the development of CAD (56). Therefore, CAD and cancer development may be associated with impairing mitochondrial function. GSEA analysis showed that *RBP1* was mainly enriched in the activation of the immunological response, regulation of cell surface receptor signaling pathways, graft immune rejection, and Epithelial-mesenchymal transition (EMT) pathways. These pathways correlate with the type of immune response in different cancers. Analysis of *RBP1* and cancer-related functional states showed that it was negatively correlated with 12 cancer-functional conditions in UM tumors and significantly positively correlated with seven cancer-functional states in lung adenocarcinoma (LUAD), including angiogenesis, quiescence, and inflammation. Angiogenesis is essential for the tumorigenesis, growth, metastasis, and dissemination of tumors (57). It has been shown that angiogenic genes can be used as biomarkers for immune infiltration and cancer development associated with LUAD in clinical settings (58). Therefore, *RBP1* may be able to be a marker for LUAD treatment as well as for assessing prognosis. In addition, DNA methylation is the most critical form of epigenetic modification (59). Analysis of the disease myth 3.0 database showed that *RBP1* was hypermethylated in CAD and 11 tumors, including PAAD, COAD, and LUAD. Epigenetic disruption of *RBP1* is a common occurrence in human cancers. In particular, the highest frequency of *RBP1* hypermethylation was observed in lymphomas and gastrointestinal tumors (60), and the expression of the *RBP1* gene is very closely related to its promoter methylation (61). The connection between four DNA methyltransferases and *RBP1* are examined. *RBP1* was associated with at least one DNA methyltransferase in multiple tumors. These results suggest that aberrant expression of *RBP1* can mediate CAD and cancer development through the regulation of DNA methylation.

In summary, *RBP1* has been identified bioinformatically as a potential essential gene that may connect CAD and cancer by way of an immune response. In a pan-cancer analysis, *RBP1* was strongly associated with immune infiltration and prognosis in different cancers and may become a new therapeutic target. In the future, developing targeted or novel immunotherapies against *RBP1* may reduce the incidence of CAD in cancer patients. However, this study was based on bioinformatics analysis, and there have not been any reported studies related to *RBP1* and CAD, more research needs to be done in follow-up experiments to identify the specific mechanisms of *RBP1* development in CAD and cancer.

## Data Availability

The datasets presented in this study can be found in online repositories. The names of the repository/repositories and accession number(s) can be found in the article/Supplementary Material.
